# Supplementary material for: Randomized Personalized Trial for Stress Management Compared to Standard of Care
Source: J Pers Med. 2026 Jan 4;16(1):23. doi: 10.3390/jpm16010023 (PMC12843166; doi:10.3390/jpm16010023)
Supplement: Supplementary file 1 [file jpm-16-00023-s001.zip › jpm-4002315-supplementary.pdf]

Supplemental Table S1. Inclusion/exclusion criteria.

**Inclusion Criteria**	**Exclusion Criteria**
Participants must meet the following criteria to be included in the study:<br>• Age ≥ 18 years<br>• English speaking<br>• Self-report of perceived stress raw score of 20 or higher using the Perceived Stress Scale (PSS)<br>• Owns and can regularly access a smartphone capable of receiving text messages and accessing the internet<br>• Can regularly wear a Fitbit device<br>• Lives in the United States<br>• ≥ 80% adherence to study measures and Fitbit wear during baseline	Persons who meet the following criteria will be excluded:<br>• Women who are pregnant<br>• Does not speak English<br>• Does not own or cannot regularly access a smartphone capable of receiving text messages<br>• Cannot regularly wear a Fitbit device<br>• Deemed unable to complete the study protocol as a result of cognitive impairment, severe medical or mental illness, or active or prior substance abuse<br>• Planned surgeries 6 months from study start date<br>• Individuals who have been previously told by a doctor to not engage in brisk walking 30 minutes, three times per week<br>• Individuals who have been previously told by a doctor to not engage in yoga<br>• Lives outside the United States

Supplemental Table S2. Descriptive Statistics for Satisfaction Measures (N=155)

Measure	Values, n (%)	All Participants	Personalized Trial Arm (N = 81)	Standard-of-care Arm (N=74)	p-value[a]
		Mean (SD; range)	Mean (SD; range)	Mean (SD; range)	
**Elements of the Personalized Trial[a]**					
1. "I found the onboarding process (from the initial survey to getting my materials) for my personalized trial straightforward and easy to follow."	155 (73)	4.39 (0.86; 1-5)	4.47 (0.76; 1-5)	4.31 (0.95; 1-5)	0.26
2. "I think my Fitbit charge 3 was easy to use."	155 (73)	4.54 (0.81; 1-5)	4.52 (0.85; 1-5)	4.55 (0.76; 1-5)	0.78
3. "The informational videos helped me understand how to participate in this study."	155 (73)	4.36 (0.83; 2-5)	4.38 (0.8; 3-5)	4.34 (0.86; 2-5)	0.74
4. "The materials I received in the mail were clear and easy to use."	155 (73)	4.47 (0.79; 2-5)	4.57 (0.77; 2-5)	4.36 (0.8; 2-5)	0.11
5. "I enjoyed receiving daily text message prompts and surveys on my cell phone."	155 (73)	3.10 (1.25; 1-5)	3.23 (1.16; 1-5)	2.96 (1.33; 1-5)	0.17
6. "I felt like I knew what was coming next in my personalized trial."	155 (73)	4.85 (1.06; 1-5)	3.95 (0.99; 1-5)	3.73 (1.14; 1-5)	0.20
7. "My personalized trial was easy to integrate into my daily routine."	155 (73)	3.54 (1.22; 1-5)	3.63 (1.2; 1-5)	3.43 (1.25; 1-5)	0.32
**Satisfaction with Components of the Trial[b]**					
1. "Your Personalized Trial for Stress Management."	155 (73)	4.12 (1.02; 1-5)	4.19 (0.87; 1-5)	4.04 (1.16; 1-5)	0.39
2. "Video explanations and demonstrations of study devices and procedures."	155 (73)	4.22 (1.01; 1-5)	4.23 (1.04; 1-5)	4.2 (0.99; 2-5)	0.85
3. "Text messaging for reminders."	155 (73)	3.77 (1.42; 1-5)	3.84 (1.36; 1-5)	3.7 (1.5; 1-5)	0.55
4. "Test messaging for survey questions."	155 (73)	3.76 (1.37; 1-5)	3.86 (1.31; 1-5)	3.65 (1.44; 1-5)	0.33
5. "Accessing the intervention videos."	155 (73)	3.99 (1.21; 1-5)	4.19 (0.95; 2-5)	3.77 (1.42; 1-5)	0.04*
6. "The yoga intervention video."	155 (73)	4.13 (1.15; 1-5)	4.16 (1.12; 1-5)	4.09 (1.18; 1-5)	0.72
7. "The mindfulness meditation intervention video."	155 (73)	4.10 (1.17; 1-5)	4.15 (1.18; 1-5)	4.04 (1.16; 2-5)	0.57
8. "The brisk walking intervention video."	155 (73)	4.10 (1.19; 1-5)	4.27 (1.01; 1-5)	3.92 (1.34; 1-5)	0.07
9. "Use of the Fitbit to track your activity and sleep."	155 (73)	4.57 (0.84; 1-5)	4.54 (0.84; 1-5)	4.61 (0.86; 1-5)	0.64
10. "Use of the study's communication over text message."	155 (73)	4.35 (1.02; 1-5)	4.38 (1.03; 1-5)	4.31 (1.02; 1-5)	0.66
11. "Presentation of your results."	155 (73)	4.33 (1.00; 1-5)	4.41 (0.92; 1-5)	4.24 (1.08; 1-5)	0.31

[a]p-values come from independent samples t-tests between the personalized and standard of care arms.
[b]Questions rated on a 5-point Likert scale from 1 "Strongly disagree" to 5 to "Strongly agree".

[c]Questions rated on a 5-point Likert scale from 1 "Not at all satisfied" to 5 "Very satisfied".

Supplemental Table S3. Participant Ratings of the Helpfulness of the Trial (N=155)

Measure	All Participants	Personalized Trial Arm (N = 81)	Standard-of-care Arm (N = 74)	p-value[a]
	N (%)	N (%)	N (%)	
**How much would you recommend this personalized trial for stress management to other persons with stress?**				**0.46**
I would not recommend	11 (7)	3 (1.9)	8 (5.2)	
I would recommend a little bit	54 (35)	32 (20.6)	22 (14.2)	
I would strongly recommend	90 (58)	46 (29.7)	44 (28.4)	
**Overall, how helpful was your participation in this study with respect to your symptoms of stress?**				**0.15**
Not at all helpful	6 (4)	2 (1.3)	4 (2.6)	
A little bit helpful	21 (13.6)	9 (5.8)	12 (7.7)	
Somewhat helpful	52 (34)	32 (20.6)	20 (12.9)	
Very much helpful	47 (30)	24 (15.5)	23 (14.8)	
Extremely helpful	29 (19)	14 (9)	15 (9.7)	

[a]p-values come from Chi-square tests between the personalized and standard of care arms.

Supplemental Figure S1. Participant Flow Diagram

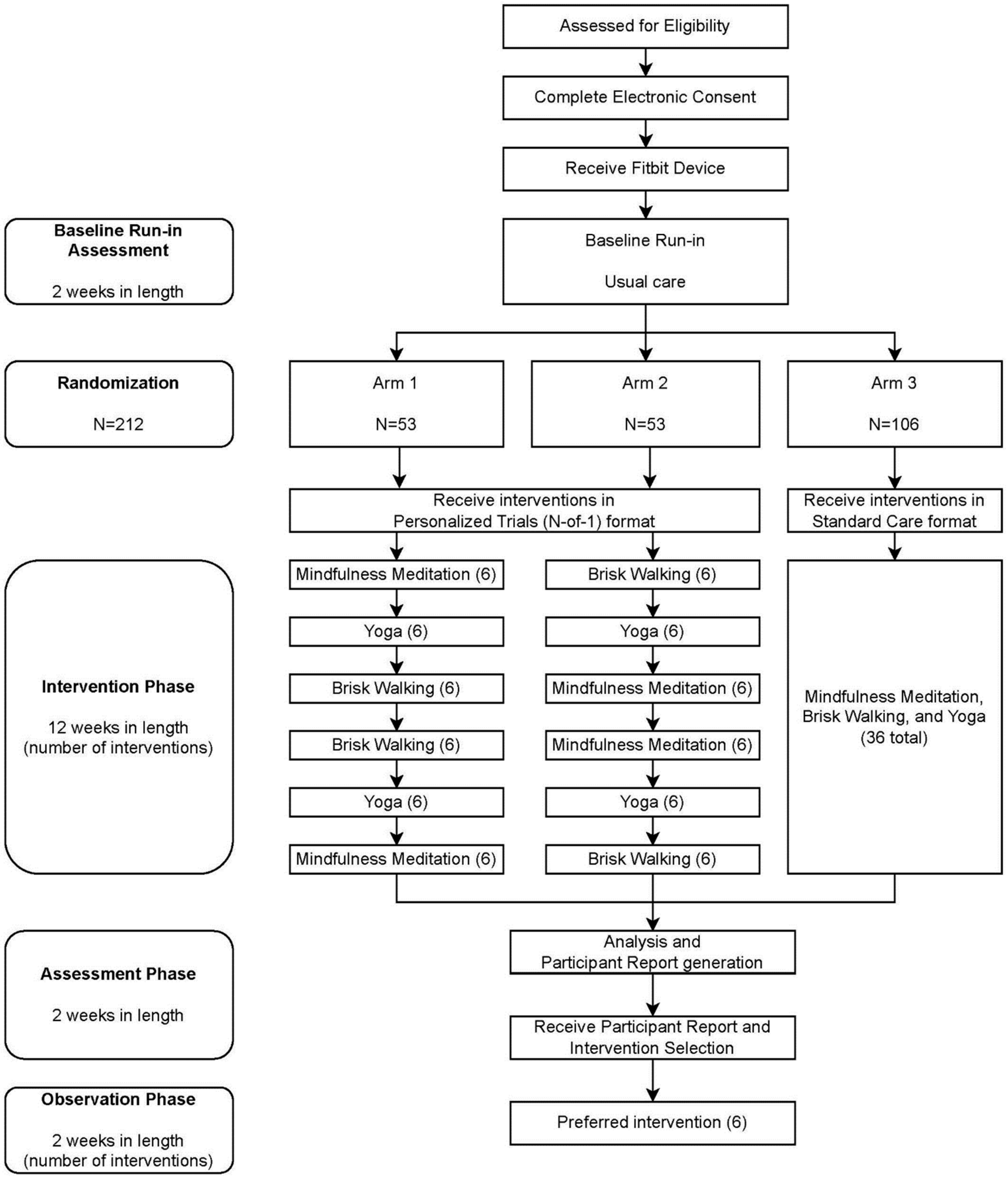

Supplemental Figure S2. Participant System Usability Scale Ratings For Each Participant

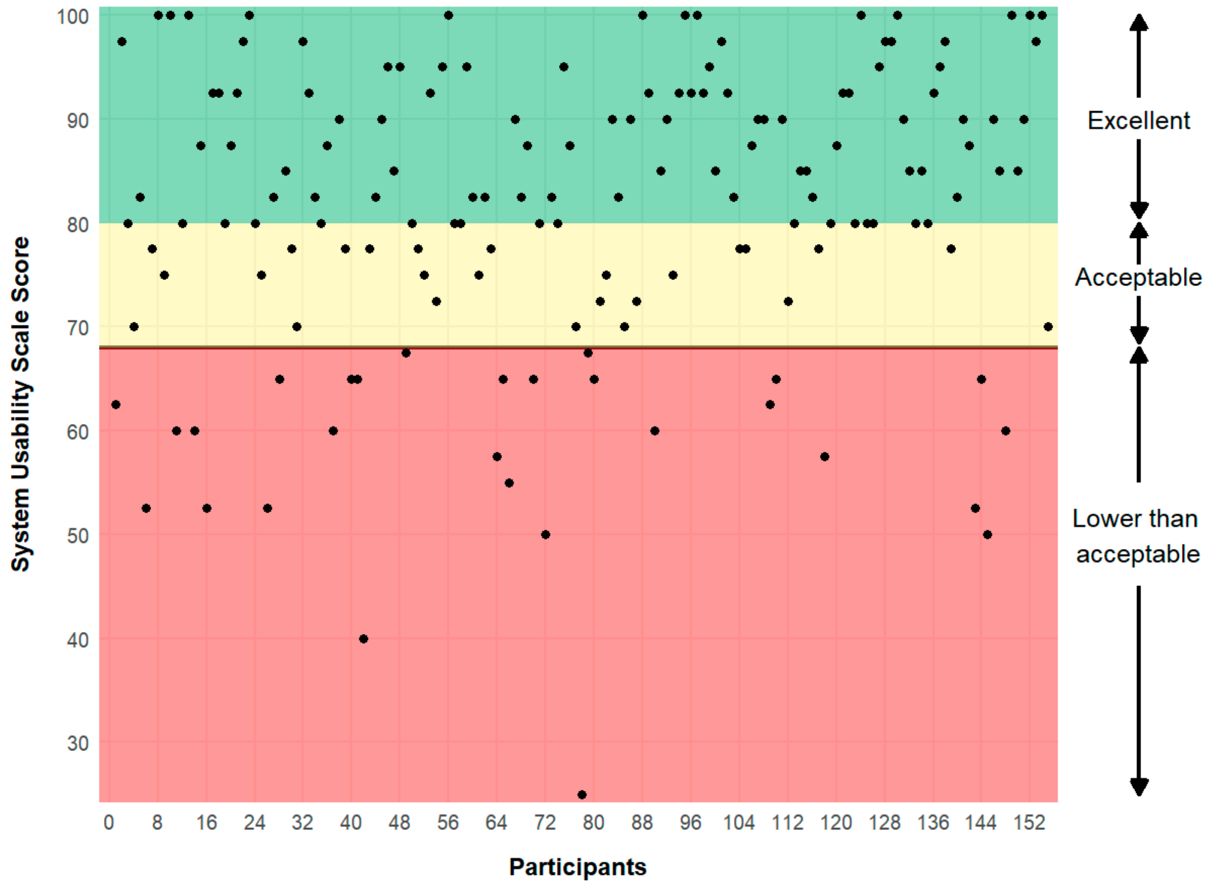

Supplemental Figure S3. Participant Satisfaction Ratings Overall and By Arm

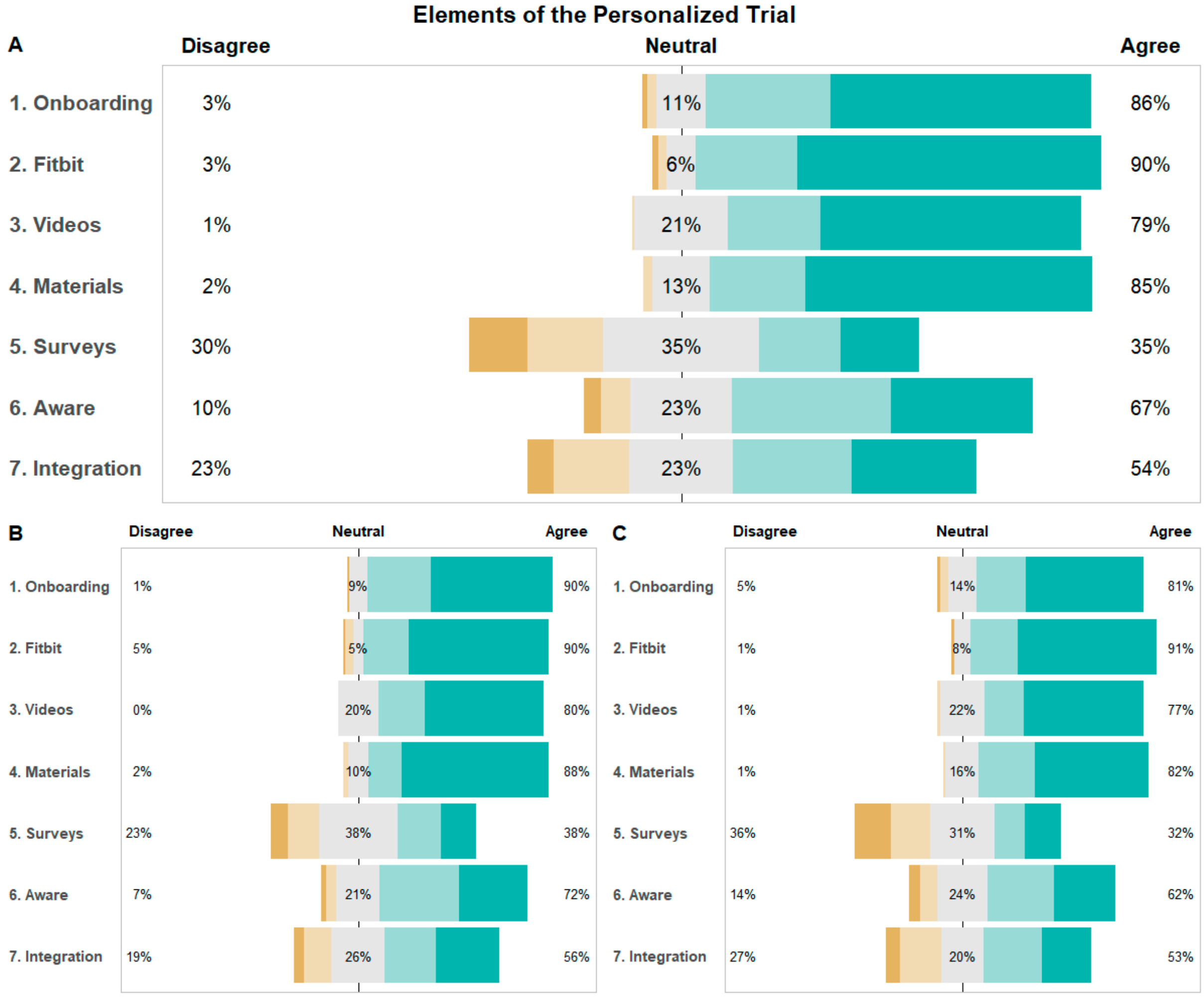

Panel A. Overall for Elements of the Personalized Trial likert plot.

Panel B. Personalized Trial Arm for Elements of the Personalized Trial likert plot.

Panel C. Standard-of-care Arm for Elements of the Personalized Trial likert plot.

Supplemental Figure S4. Participant Satisfaction Ratings Overall and By Arm

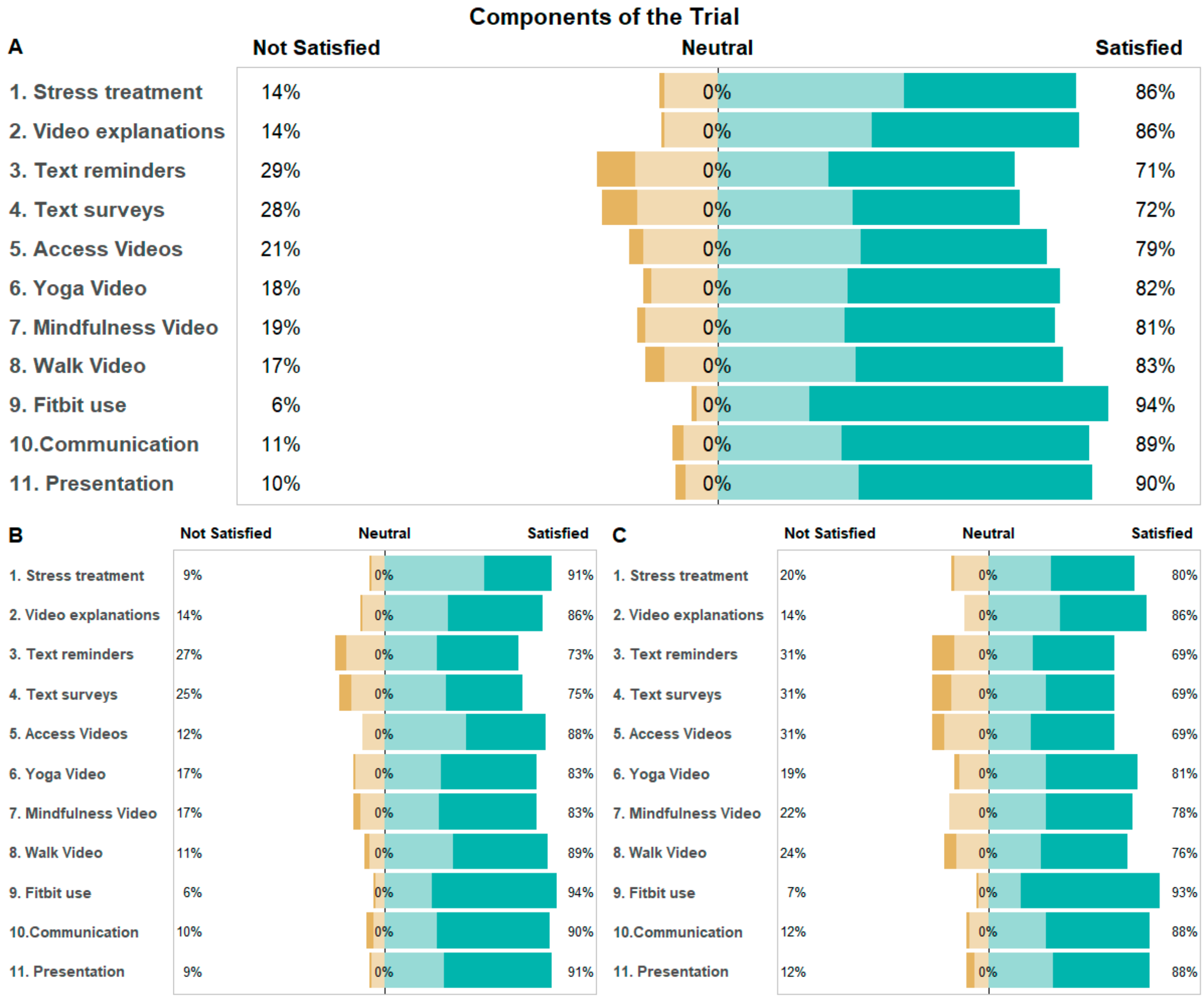

Panel A. Overall Satisfaction in Components of the Trial likert plot.

Panel B. Personalized Trial Arm Satisfaction in Components of the Trial likert plot.

Panel C. Standard-of-care Arm Satisfaction in Components of the Trial likert plot.
